# Management of a Persistent Radicular Cyst in the Maxillary Right Lateral Incisor: A Case Report

**DOI:** 10.7759/cureus.66421

**Published:** 2024-08-08

**Authors:** Shaswatee Panda, Abhisek Das, Yoshaskam Agnihotri, Sambarta Das, Esha Bhagat

**Affiliations:** 1 Department of Conservative Dentistry and Endodontics, Hi-Tech Dental College & Hospital, Bhubaneswar, IND

**Keywords:** radicular cyst, prf membrane, bone graft, apicoectomy, mta obturation

## Abstract

A 32-year-old male patient presented with a chief complaint of ongoing endodontic treatment and swelling in the hard palate, specifically in the rugae region. On examination, a soft, non-tender, non-fluctuant swelling was observed between teeth #11 and #12, accompanied by radiographic evidence of periapical radiolucency and perforation. Cone beam computed tomography (CBCT) scans confirmed a well-defined radiolucency in the affected region, indicative of a radicular cyst. Root canal treatment was completed with mineral trioxide aggregate (MTA) obturation during the second visit. An apicoectomy was performed to remove the cystic content, followed by the placement of bone graft material, a platelet-rich fibrin (PRF) membrane, and sutures to facilitate bone regeneration. This comprehensive approach aimed to resolve the periapical pathology and promote tissue healing around the affected tooth.

## Introduction

Cysts in the jaws are a common pathological finding, with the majority originating from odontogenic epithelium [1]. These cysts are broadly classified into two categories: inflammatory and developmental cysts. Inflammatory cysts, such as radicular cysts, are primarily associated with non-vital or pulpally involved teeth and are believed to originate from the epithelial rests of Malassez [1,2]. These epithelial remnants, part of the periodontal ligament, proliferate in response to inflammatory stimuli, leading to cyst formation [3].

Accurate diagnosis and management of these lesions are crucial for successful treatment outcomes [4]. Histopathological examination remains the gold standard for definitive diagnosis, providing detailed information about tissue composition, cellular characteristics, and any atypical features. This microscopic analysis allows for the accurate identification of cystic lesions and aids in differentiating them from other pathologies, such as odontogenic tumors, which may have similar radiographic appearances but vastly different treatment protocols and prognoses [5].

Advanced radiological techniques, such as cone beam computed tomography (CBCT), have revolutionized the diagnostic approach to maxillofacial pathologies. CBCT offers three-dimensional imaging with high spatial resolution and lower radiation exposure compared to traditional CT scans [6]. It provides detailed information about the extent of the lesion, its relationship to adjacent anatomical structures, and the integrity of cortical plates, crucial factors in treatment planning.

This case report presents the management of a persistent radicular cyst associated with the maxillary right lateral incisor (tooth #12) in a patient dissatisfied with previous treatment outcomes. The case highlights the importance of a multidisciplinary approach, incorporating advanced diagnostic tools such as CBCT, histopathological examination through biopsy, and innovative treatment modalities such as biomimetic restorative materials, bone grafts, and platelet-rich fibrin (PRF) membranes.

Biomimetic materials, designed to mimic natural tissue properties, have shown promise in endodontic and restorative dentistry. These materials, such as bioactive cement and ceramics, can enhance the healing process by promoting tissue regeneration and integration. Similarly, bone grafts and PRF membranes contribute to faster and more predictable healing in surgical procedures [7].

Bone grafts, whether autogenous, allogenic, or synthetic, provide a scaffold for new bone formation and help maintain the alveolar ridge contour. PRF, a second-generation platelet concentrate, contains a dense fibrin matrix rich in growth factors and cytokines [8]. When used as a membrane, PRF promotes soft tissue healing, reduces postoperative pain, and accelerates bone regeneration [9].

The synergistic use of these advanced diagnostic and therapeutic modalities not only addresses the pathological lesion but also focuses on restoring form and function with minimal morbidity [10]. This case report aims to elucidate the successful endodontic and surgical management of a persistent inflammatory cyst, emphasizing the role of a comprehensive, evidence-based approach in achieving favorable outcomes, even in cases where previous treatments have failed to provide satisfactory results.

## Case presentation

**Figure 1:**
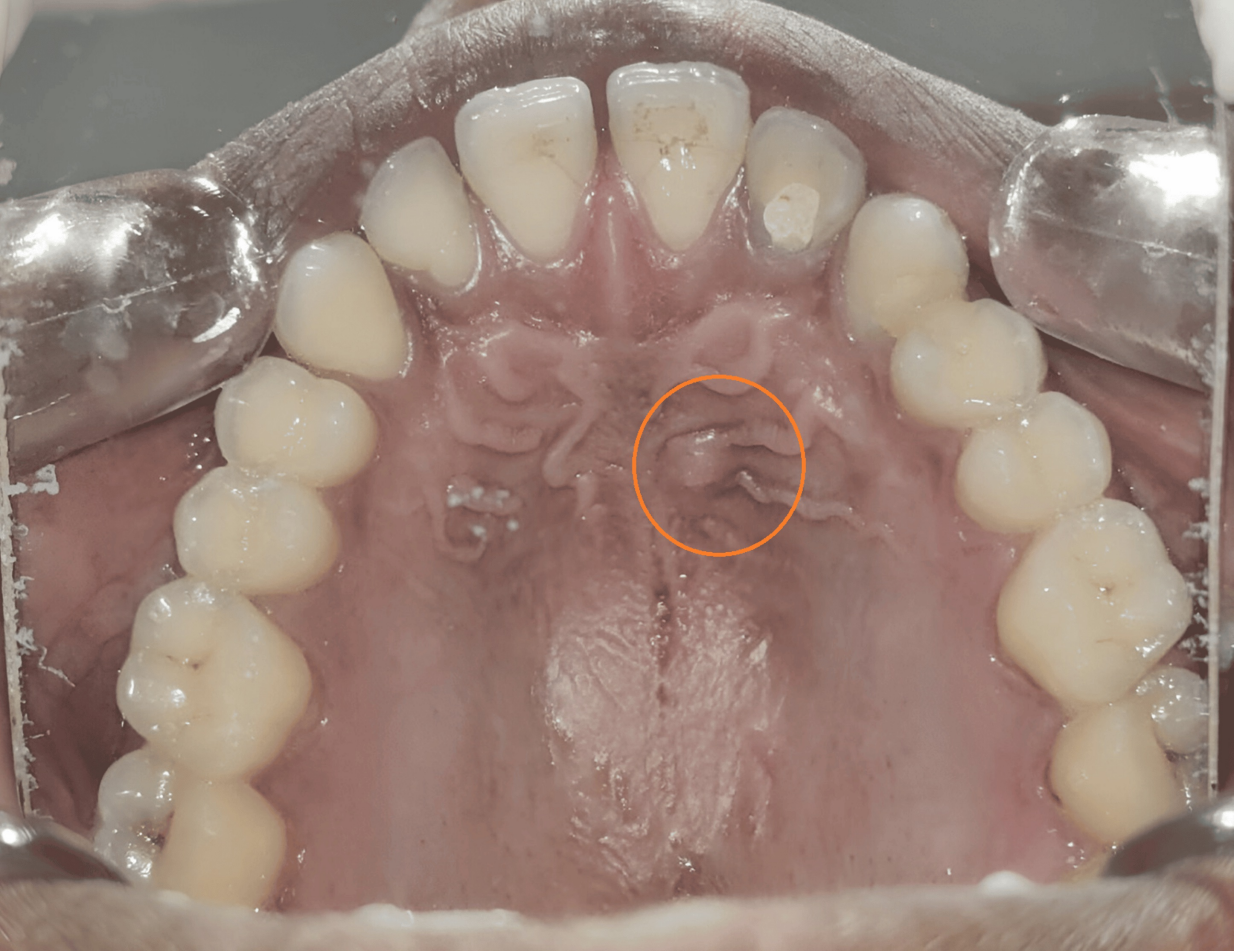
Pre-operative intraoral photograph showing palatal swelling

A 32-year-old male patient presented with a complaint of ongoing endodontic treatment for the past year and swelling in the palate. The swelling was located in the hard palate, specifically in the rugae region (Figure 1), and was soft and non-fluctuant. The patient had no relevant medical or drug history. His dental history included root canal treatment on tooth #13 and ongoing treatment on tooth #12. Extra-oral examination revealed no swelling, lymph node enlargement, or sinus drainage. Intra-oral examination showed localized swelling in the hard palate between teeth #11 and #12, measuring approximately 3 mm x 4 mm. The swelling was non-tender, soft, non-fluctuant, and non-persistent, with no sinus drainage observed. 

There was no mobility observed for tooth #12. A periodontal pocket of around 4 mm was recorded around tooth #12.

**Figure 2:**
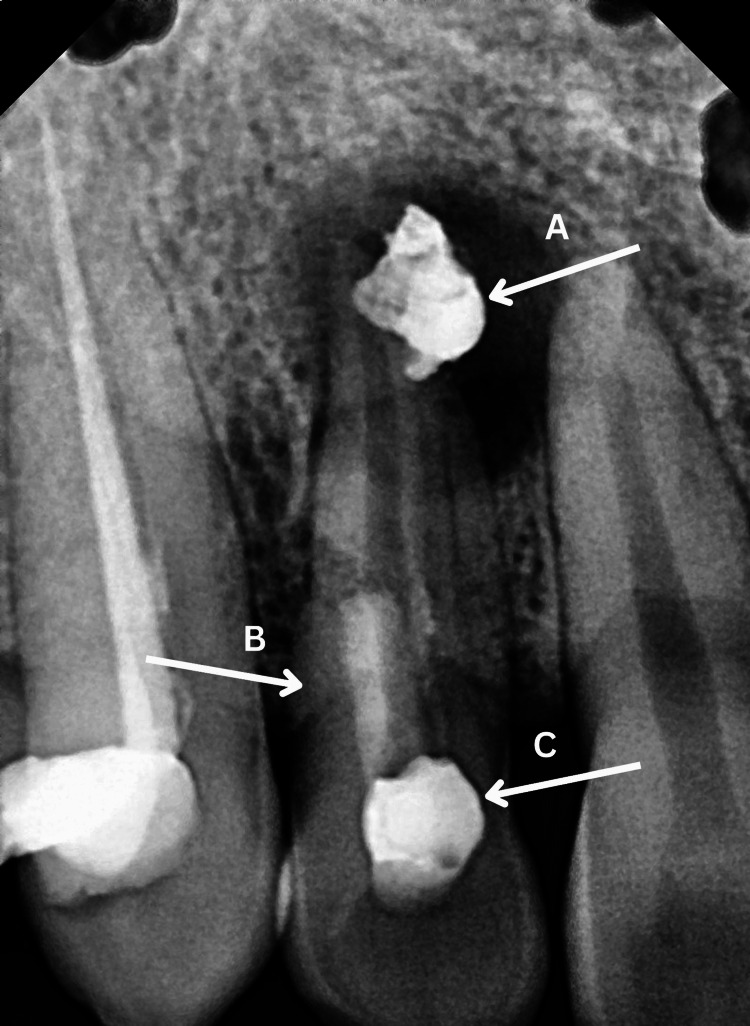
Pre-operative RVG (A) Extrusion of expected intracanal medicament, (B) expected root perforation, and (C) lateral shift of access opening RVG: Radiovisiography

Radiographic examination revealed loss of lamina dura and periodontal ligament (PDL) widening around tooth #12, along with a well-defined periapical radiolucency. The access opening had a lateral shift, and mild radiopacity was observed extending from the cervical area to the coronal third of the root, indicative of a perforation. No hard-tissue fracture was noted. A radiopaque foreign object near the apical region of the root suggested the presence of perforation repair material or extrusion of intracanal medicament (Figure 2).

**Figure 3:**
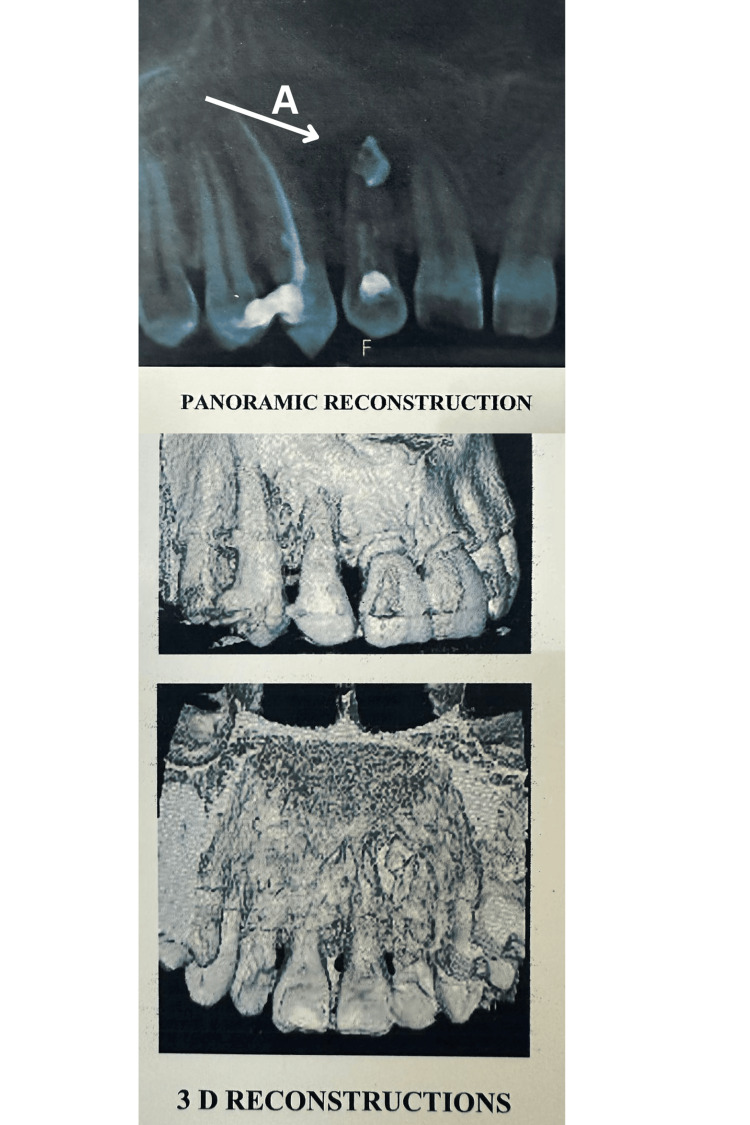
Panoramic reconstruction view and three-dimensional view of CBCT i.r.t 12 A: Periapical Radiolucency

**Figure 4:**
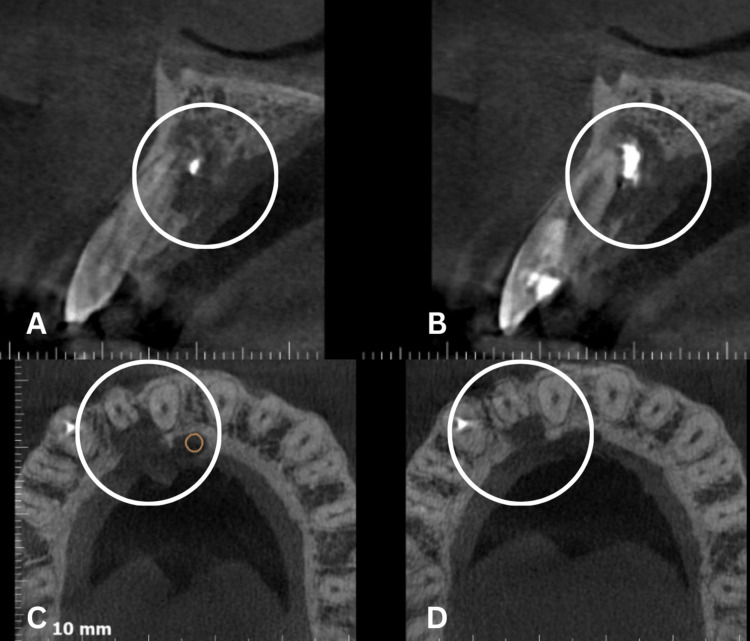
Pre-operative CBCT image A: Pre-operative cross-sectional CBCT scan demonstrating the extent of the lesion. B: Pre-operative cross-sectional CBCT scan showing another view of the lesion. C: Pre-operative axial CBCT scan highlighting the lesion. D: Pre-operative axial CBCT scan providing another view of the lesion.

CBCT reports indicated a well-defined radiolucency in the region of tooth #12, measuring approximately 11 x 10 mm (Figures 3-4). The lesion had perforated the palatal cortical plate in the region of tooth #12. A well-defined radiopacity, similar in density to restorative material, was observed superimposed on the root apex of tooth #12. Extrusion of filling material, measuring approximately 5 x 3 mm, was evident outside the root apex. No evidence of root resorption of the involved teeth was noted. Histopathological examination confirmed the diagnosis of an inflammatory(radicular) cyst associated with tooth #12. The histopathological report is attached as a supplementary file in the appendices section for reference.

During the first visit, under rubber dam isolation, the temporary restoration on tooth #12 was removed, and the working length was determined using a #20k file. Cleaning and shaping was performed up to an F5 file, followed by irrigation with 2% chlorhexidine. Calcium hydroxide was used as an intracanal medicament.

**Figure 5:**
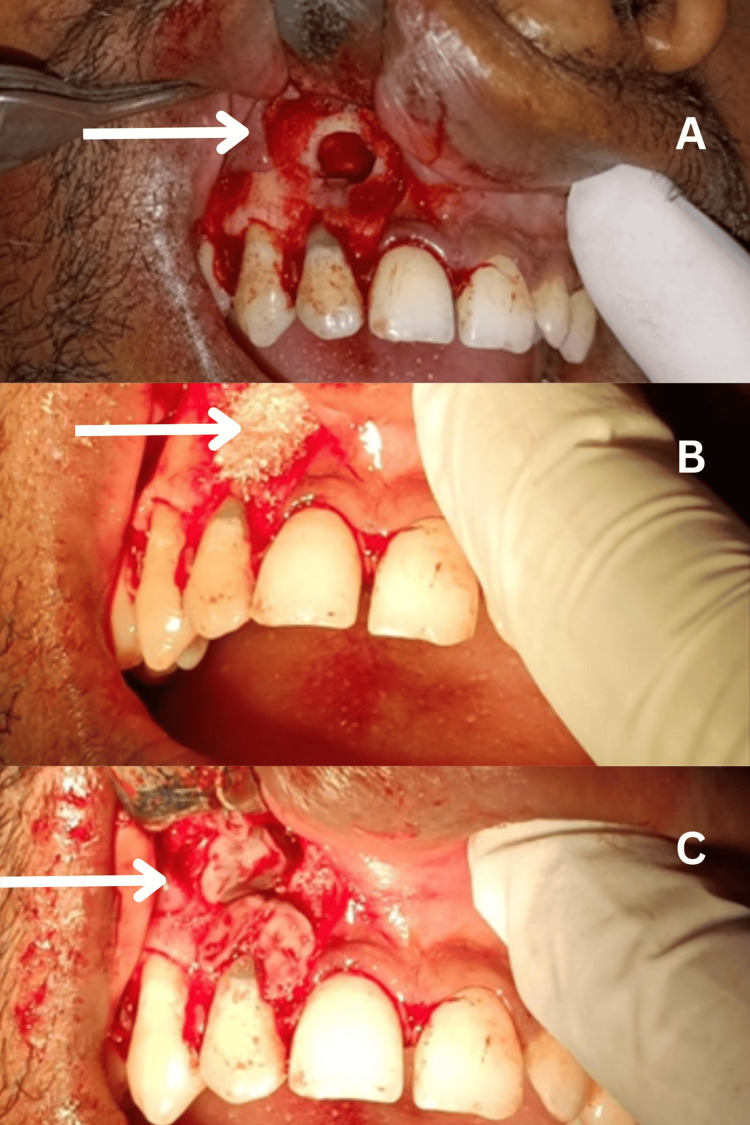
Intraoperative images showing the surgical procedures A) Post-apicoectomy procedure; (B) bone graft placement; and (C) placement of PRF membrane

In the second visit, obturation of tooth #12 was performed using mineral trioxide aggregate (MTA), followed by the placement of moist cotton and temporary restoration. The patient was recalled after 48 hours for an apicoectomy on tooth #12. The cystic content was removed, and an apicoectomy was performed. Synthetic bone graft material (Bio-Oss) was placed, followed by the placement of an A-PRF membrane and sutures (simple interrupted and vertical mattress using Prolene) (Figure 5).

**Figure 6:**
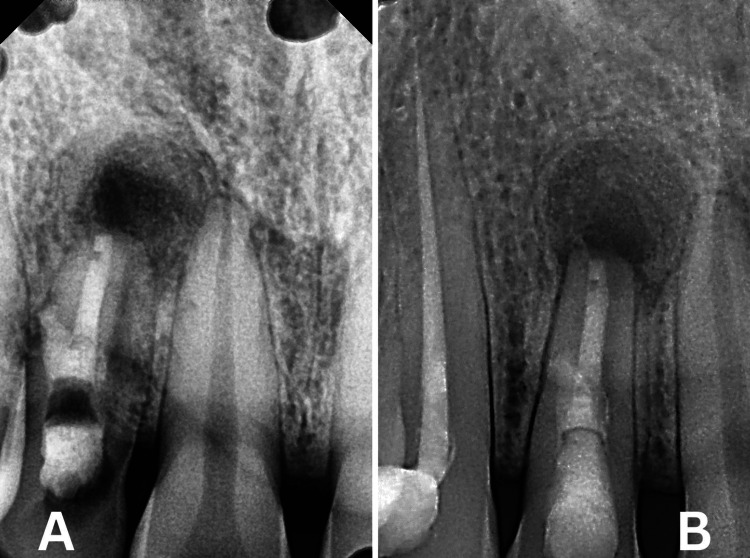
Post-operative RVG at three-month and six-month follow-up A) Three-month follow-up; B) Six-month follow-up RVG: Radiovisiography

The patient was prescribed postoperative antibiotics three times a day for five days and analgesics twice a day for five days. The patient was recalled for follow-up reviews at one-month, three, and six-month intervals (Figure 6).

## Discussion

Iatrogenic errors during endodontic treatment, such as exaggerated or misguided access cavities, can lead to root perforations, compromising the tooth's structural integrity and increasing the risk of coronal or radicular fractures [10]. Endodontists frequently face complex challenges, including root perforations, overfilling, endodontic-periodontal lesions, root fractures, persistent periapical biofilms, traumatic dental injuries, instrument fractures, apical periodontitis, and root resorption [11]. These factors, either individually or in combination, can significantly impact the prognosis, making it doubtful or poor. Close post-surgery follow-up is essential for radicular cysts to ensure proper bone regeneration [12].

In this case, a coronal root perforation was identified. The management of such perforations has evolved with the introduction of bioactive materials such as MTA. The MTA has emerged as the preferred material for perforation repair due to its superior sealing ability, biocompatibility, and ability to promote healing. Repairing with MTA involves identifying the involved area and applying the cement in a thickness that provides an adequate seal to promote repair and healing [13].

The MTA was used in this case because of its unique properties. It exhibits excellent sealing ability even in moist environments, a common scenario at perforation sites. Its good marginal adaptation minimizes leakage, while its high biocompatibility promotes a favorable tissue response. Furthermore, MTA has demonstrated long-term success in managing teeth with large periapical lesions when used as a root canal filling material. Given that one of the primary objectives of endodontic therapy is to prevent reinfection of the root canal system, MTA's antimicrobial properties and ability to induce hard tissue formation make it an invaluable material [14,15].

Bone grafting was used in this case to accelerate the healing process and significantly improve bone quality. Synthetic bone grafts, with their interconnected porous structure mimicking cancellous bone, offer several advantages. Their resorbable nature allows for better vascularization and increased surface area for cell attachment, leading to faster healing and effective bone formation [16]. The predictable resorption rate ensures gradual replacement by native bone, promoting seamless integration with the surrounding tissues [17]. Additionally, their radiopacity facilitates easy monitoring of bone healing on radiographs [18].

PRF, a second-generation platelet concentrate, was employed alongside the bone graft in the present case to enhance healing and improve prognosis. PRF is a rich source of growth factors, including platelet-derived growth factor (PDGF), transforming growth factor-β1 (TGF-β1), and insulin-like growth factor (IGF) [19]. These growth factors play pivotal roles in cell migration, attachment, proliferation, and differentiation, thereby accelerating and modulating the healing process [20]. In endodontics, PRF has shown promise in pulp-dentin complex regeneration, underscoring its potential to promote tissue repair.

Potential complications, such as fluid accumulation in the palatal area, risk of damaging anatomical structures, and ensuring proper healing, were considered [10,11]. However, no complications were encountered during the surgery.

## Conclusions

This case report is a witness to the successful interdisciplinary dental treatment of a chronic inflammatory cyst in the maxillary right lateral incisor. A coordinated effort involving multiple dental specialties, combined with newer diagnostic techniques, and appropriate surgical and non-surgical interventions, leads to successful treatment. A favorable outcome was achieved by using materials such as MTA, synthetic bone grafts, and PRF membranes. Managing such complex lesions, especially in the presence of iatrogenic complications such as root perforations, requires a high level of clinical expertise, meticulous decision-making, and appropriate use of technologies.

## Appendices

**Table 1:** Histopathological report

Field	Details
Department	Department of Oral & Maxillofacial Pathology
Age/Sex	32/M
Received Date	1.2.2023
Reporting Date	7.2.2023
Clinical Diagnosis	Not provided
Specimen	Incisional
Gross	Multiple bits of tissue, blackish, soft measuring about 8 mm x 5 mm. NGS
Microscopy	H & E stained section shows bony areas, fibrous connective tissue stroma infiltrated with chronic inflammatory cells and stratified squamous epithelium. Hemorrhagic areas are present.
Diagnosis	Inflammatory Cyst
